# Predictive Value of Radiographic Tumor Burden Score in Hepatocellular Carcinoma Within Milan Criteria After Microwave Ablation: Implications for Long‐Term Outcomes and Treatment Planning

**DOI:** 10.1002/cam4.70806

**Published:** 2025-04-24

**Authors:** Xiaolin Liu, Jing Wang, Feng Xu, Jing Chen, Mingyuan Zhu, Xiaoguang Wang

**Affiliations:** ^1^ Department of Hepatobiliary and Pancreatic Surgery The Second Affiliated Hospital of Jiaxing University Jiaxing China; ^2^ Department of Digestive Endoscopy The General Hospital of Northern Theater Command Shenyang China; ^3^ Department of Hepatobiliary and Splenic Surgery Shengjing Hospital of China Medical University Shenyang China

**Keywords:** hepatocellular carcinoma, microwave ablation, prognosis, propensity score matching, tumor burden score

## Abstract

**Objective:**

This study aimed to investigate the predictive value of the radiographic tumor burden score (TBS) for long‐term outcomes in hepatocellular carcinoma (HCC) patients meeting Milan criteria after microwave ablation (MWA) and to delineate its significance in guiding treatment planning.

**Methods:**

Retrospective analysis was conducted on clinical data from 198 HCC patients meeting Milan criteria, who underwent MWA at our hospital from January 2011 to December 2018. Using X‐tile software, the optimal critical value of TBS was determined, leading to the categorization of patients into high‐ and low‐TBS groups. Propensity score matching (PSM) was applied to balance covariates between these groups.

**Results:**

Before PSM, the 5‐year overall survival (OS) rate and recurrence‐free survival (RFS) rate in the high‐TBS (47 cases) and low‐TBS groups (151 cases) were 32.8% versus 59.7% (*p* = 0.033) and 23.4% versus 50.9% (*p* = 0.016), respectively. Following PSM, the 5‐year OS rate and RFS rate in the high‐TBS (44 cases) and low‐TBS groups (95 cases) were 30.2% versus 64.1% (*p* = 0.011) and 21.9% versus 45.9% (*p* = 0.0059), respectively. Cox analysis identified high TBS and percutaneous microwave ablation (PMWA) as independent risk factors for OS and RFS. The stratified analysis revealed that the median RFS time for patients undergoing laparoscopic microwave ablation (LMWA) (20 cases) and PMWA (24 cases) in the high‐TBS group (44 cases) was 45 and 10.5 months, respectively (*p* = 0.006).

**Conclusion:**

TBS emerged as a robust predictor for the long‐term outcomes of HCC within Milan criteria after MWA. A higher TBS was associated with a diminished long‐term survival rate. Notably, among HCC patients meeting Milan criteria, those with TBS > 3 exhibited a prolonged median RFS time following LMWA compared to PWMA.

## Graphical abstract

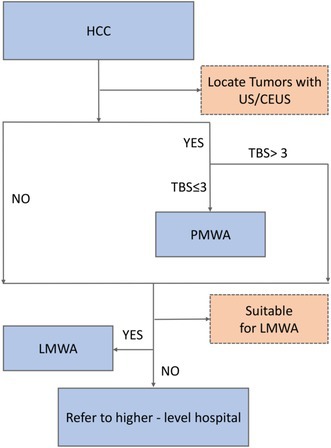

## 1 Introduction

Hepatocellular carcinoma (HCC) is the most prevalent primary hepatic malignancy, characterized by a considerable mortality rate. While surgical resection remains the most effective treatment for HCC, its inherent trauma and heightened risk of complications necessitate exploration of alternative methods. Microwave ablation (MWA) has emerged as a promising treatment due to its advantages, including low trauma, high safety, and precise localization [1, 2, 3].

However, the diverse prognostic outcomes observed in HCC patients treated with MWA, attributed to variations in tumor morphology, underscore the critical need for more accurate prognostic tools [4]. Existing evaluation systems, relying on bivariate variables like tumor size and number, such as the Barcelona Clinic Liver Cancer (BCLC) staging system, have exhibited limitations in statistical power and accuracy. The “subway ticket model”, proposed by Mazzaferro et al., correlates survival rates with the diameter and number of tumors [5]. Additionally, models based on total tumor diameter and volume have been used but are not without drawbacks [6, 7]. Sasaki et al. pioneered the radiographic TBS model, utilizing tumor diameter and number as continuous variables to evaluate the prognosis of patients with colorectal liver metastasis, demonstrating its effectiveness [8]. Subsequently, TBS has found applications in assessing liver cancer survival and stratifying prognostic studies for various treatments, including hepatectomy, liver transplantation, and nonsurgical treatment [9, 10, 11], indicating its potential as a reliable proxy indicator of tumor burden.

Despite its successful application in diverse scenarios, the accuracy of TBS in predicting the prognosis of MWA treatment in HCC remains unclear. This study employs propensity score matching (PSM) analysis to evaluate the predictive value of TBS for long‐term outcomes in HCC patients meeting Milan criteria after MWA. Additionally, we elucidate the significance of TBS in guiding treatment planning.

## 2 Materials and Methods

### 2.1 Patients

**FIGURE 1:**
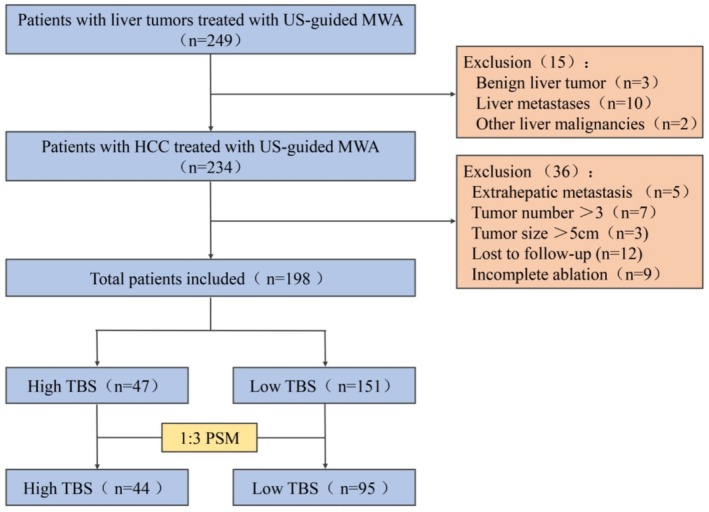
Flow chart of patient selection. HCC, hepatocellular carcinoma; MWA, microwave ablation; PSM, propensity score matching; TBS, tumor burden score; and US: ultrasound.

This retrospective study received approval from the medical ethics committee of the Second Affiliated Hospital of Jiaxing University (2023SW001‐01). The study focused on HCC within Milan criteria after MWA, encompassing clinical data collected from January 2011 to December 2018. Key variables included age, sex, body mass index (BMI), alpha‐fetoprotein (AFP) levels, presence of hepatitis B virus (HBV), cirrhosis, portal hypertension, Child–Pugh grade, tumor diameter, tumor number, ablation method, and the tumor's proximity to major blood vessels or the subcapsular region of the liver. Inclusion criteria were as follows: (a) initial HCC onset without prior treatment, (b) preoperative Child–Pugh classification of liver function Grade A or B, (c) absence of distant metastasis, vascular invasion, and lymphatic metastasis, (d) maximum diameter of a single tumor ≤ 50 mm or ≤ 3 tumor nodules with a maximum diameter ≤ 30 mm, (e) confirmation of complete ablation by imaging examination within 1 month after MWA, and (f) availability of complete patient data and follow‐up. Exclusion criteria were as follows: (a) a history of other malignant tumors, (b) severe complications, and (c) a platelet count < 30 × 10^9^/L. After screening, 198 patients fulfilling the inclusion criteria were included in the study. All the cases analyzed herein had complete data without any missing values. The data were meticulously collected and organized following the research protocol and data management plan. Any cases with missing or incomplete data were excluded to guarantee the robustness and reliability of the analysis results (Figure 1).

### 2.2 MWA Equipment and Procedure

**FIGURE 2:**
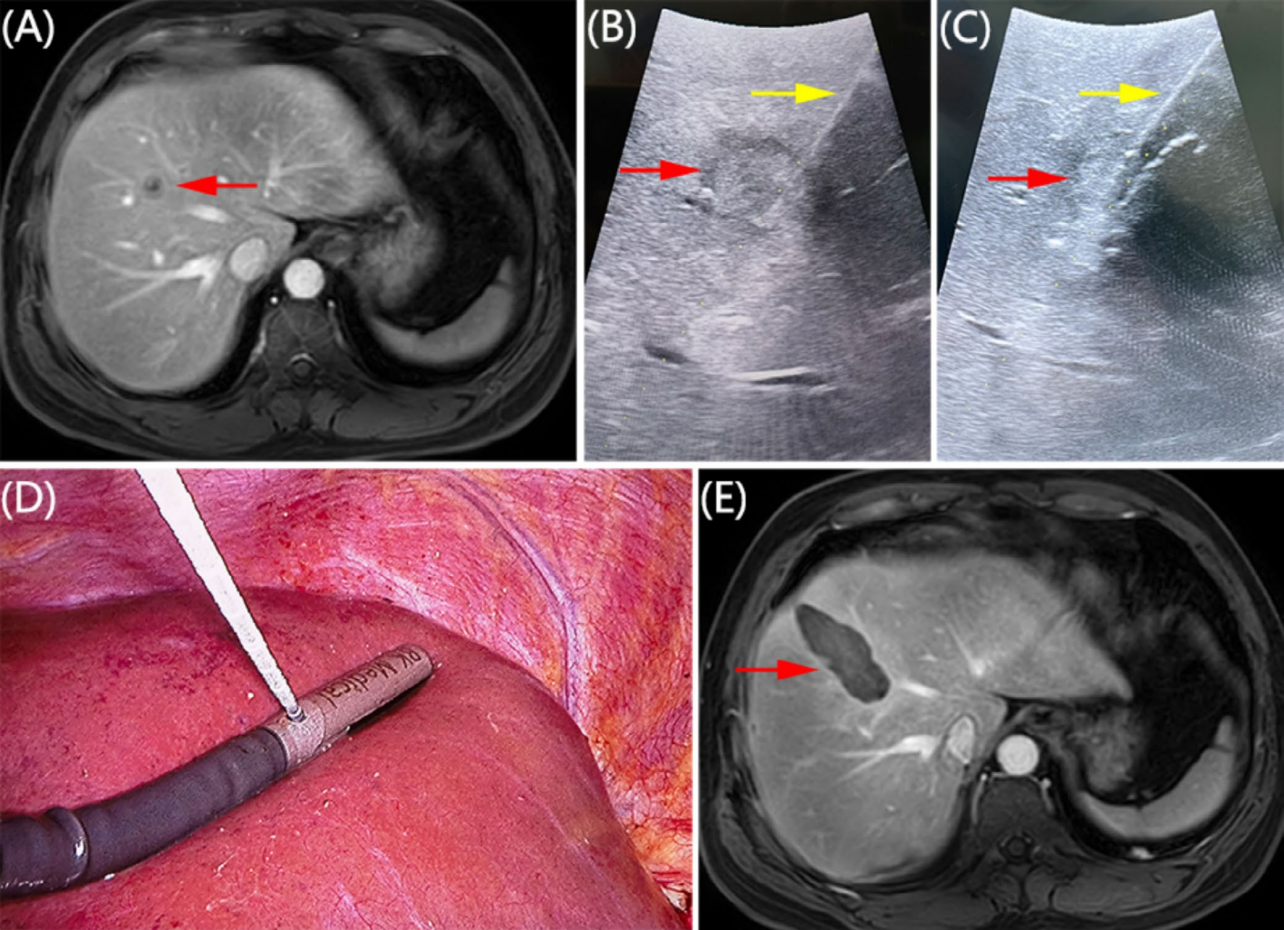
Example of using ultrasound‐guided MWA for treating HCC are located in the Junction of Segments 5 and 8 of liver (adjacent to middle hepatic vein). (A) A single nodule (indicated by the red arrow) was visible on the preoperative MRI scan. (B and C) The needle (yellow arrow) was placed at the left and right side of the tumor center because the nodule(red arrow) was 2.1 cm in diameter and adjacent to the middle hepatic vein. (D) Insertion of the antenna into the nodule was conducted under laparoscopic ultrasound guidance. (E) The tumor was entirely ablated, as evidenced by the postoperative MRI scan.

The MWA equipment employed in this study was the Nanjing YIGAO MWA system (ECO‐100AI10, Nanjing YIGAO Medical, China) along with its compatible unipolar MWA needle (2.0 mm diameter, 14 gauge, length 20 cm). The procedure was conducted collaboratively with experienced hepatobiliary and ultrasound specialists, each possessing 5–10 years of experience in MWA. Tumor lesions were identified using contrast‐enhanced ultrasound (CEUS), enhanced computed tomography (CT), or magnetic resonance imaging (MRI) scans performed prior to MWA (Figure 2A). Real‐time ultrasonic positioning guided the MWA needle toward the tumor location (Figure 2D). The needle's position was adjusted based on the lesions' size and location. Taking into account the constraints associated with percutaneous microwave ablation, including the potential for hemorrhage, the discomfort experienced with local anesthesia, the depth of tumor positioning, and the vicinity to blood vessels or crucial organs, opting for laparoscopic ultrasound‐guided microwave ablation may be a more suitable alternative. For tumors less than 2 cm in diameter, the needle was centrally placed. For nodules with a diameter of 2–3 cm, the needle was placed at the left and right sides of the tumor center (Figure 2B,C). Multiple overlapping ablations, achieved by repositioning the antenna, were necessary for nodules with a diameter greater than 3 cm. The route of multiple punctures was preset before ablation to minimize the impact of hyperechogenic gas [3]. A waiting period of 5–10 min was observed between two punctures to allow gasification to subside. The ablation parameters included a transmitting power of 60 W and a duration of 180 s. Each puncture site underwent two cycles of ablation [3]. The number of ablations was determined based on various factors such as tumor size, degree of destruction, and extent of ablations. Complete ablation was confirmed when the ideal range was reached without any observable residual tumor. After ablation, MWA continued to coagulate the needle path to prevent bleeding and seeding. A subsequent ultrasound scan was performed to check for complications such as bleeding, pneumothorax, or fluid buildup. Patients were advised to rest in bed for 24 h after the ablation procedure. Enhanced CT, enhanced MRI (Figure 2E), or CEUS was re‐examined 5–7 days after ablation to assess the need for secondary ablation based on tumor inactivation. If the ablation edge was larger than 0.5 cm from the original tumor, it was considered complete ablation, and local enhancement in the tumor area indicated residual tumor.

### 2.3 Calculation of TBS and Definition of Optimal Cut Point

**FIGURE 3:**
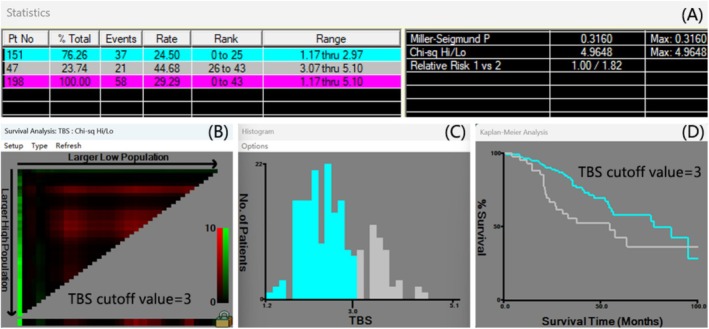
Identification of the cut‐off points produced by the X‐tile plot. The prognostic power was strongest when the cut‐off value of TBS was 3 (A–D).

Preoperative imaging provided the maximum tumor diameter and the number of tumors, with tumor size determined by the size of the largest lesion in the case of multiple nodules. The TBS was calculated based on the maximum tumor diameter and the number of tumors obtained from preoperative imaging [9, 12]. Specifically, the formula was TBS^2^ = (number of tumors)^2^ + (maximum tumor diameter)^2^. When determining the TBS, we took the maximum tumor diameter as the variable on the *x*‐axis and the number of tumors as the variable on the *y*‐axis for the calculation. For instance, if a patient had two tumors and the maximum tumor diameter was 3 cm, then its TBS^2^ = 2^2^ + 3^2^ = 13, and the TBS was approximately 3.6. The X‐tile software, developed by Camp at Yale University, emerged as one of the initial applications to identify the optimal threshold for tumor prognostic indicators [13]. In this study, we adopted X‐tile software to confirm that the optimal cut‐off point for TBS OS was 3 by minimum *p*‐value from log‐rank χ^2^ test, based on which patients were divided into two groups with the strongest discriminatory capacity (Figure 3). Consequently, patients were categorized into two groups: low‐TBS (151 cases) (TBS ≤ 3) and high‐TBS groups (47 cases) (TBS > 3). Figure 3 shows the distribution of TBS values in our patient cohort and clearly indicate the selected cut‐off point (TBS = 3) along with the corresponding survival curves for the two groups (TBS ≤ 3 and TBS > 3) generated by the Kaplan–Meier method. This will allow readers to have a more intuitive understanding of how the cutoff was determined and its impact on patient survival.

### 2.4 PSM Analysis

To reduce confounding bias, we performed a PSM analysis on all prognosis‐affecting clinical factors, excluding TBS as it is our main research variable. Given the large sample size gap between high‐ and low‐TBS groups, we used the 1:3 nearest‐neighbor matching method. With TBS as the dependent variable and other prognostic factors as covariates, a logistic regression model (caliper value = 0.1) calculated propensity scores for matching similar ‐score cases. During this, continuous variables like age and BMI were turned into categorical ones according to their prognostic impacts and included in the model for score calculation. Categorical variables such as gender and HBV infection status were added as dummy variables. After matching, confounding factors were balanced between the two groups, reducing interference with results. For example, the significant difference in ablation methods before matching (*p* < 0.05) disappeared after PSM (*p* > 0.05), showing PSM's effectiveness in balancing confounding factors.

### 2.5 Sample Size

We used 5‐year overall survival after MWA as an outcome measure to assess the feasibility of the current sample size. Using Pass 2021 (NCSS, Kaysville, Utah, U.S.) statistical software, the α‐error was set at 0.05 and the power at 1‐β = 0.80. According to previous data [14], we estimated that the 5‐year overall survival rate of patients with TBS ≤ 3 would have been 80% based on an assumed difference of 25% between the two groups. According to a 1:3 matching ratio, the sample sizes for the high‐TBS group and the low‐TBS group were calculated to be 34 and 102 cases, respectively. In this study, the final number of cases in the two groups was 44 and 95, respectively, making the sample size feasible.

### 2.6 Follow‐Up Contents

All patients underwent close monitoring and followed up at the outpatient department until December 2019, with the follow‐up period ranging from 0.5–105 months. The median follow‐up time was 29.7 months, and no cases were lost to follow‐up. Tumor status was observed through re‐examinations, including enhanced CT, MRI, or CEUS 1 month after MWA and every 3 months thereafter. The presence of local enhancement in the tumor area indicated recurrence.

### 2.7 Statistical Analysis

The optimal critical value of TBS was determined using the Kaplan–Meier module of X‐tile 3.6.1 software (https://x‐tile.software.informer.com/). Analysis was conducted using SPSS 29.0, with statistical charts generated using SPSS 29.0. Continuous variables were compared using independent‐sample *t‐tests* or nonparametric tests, while categorical variables were analyzed using the chi‐square test. Measurement data were presented as (mean ± standard deviation), counting data as frequency, and the survival rate was calculated using the Kaplan–Meier method. Univariate and multivariate analyses were performed using the Cox model, and forest maps were constructed. A *p‐*value < 0.05 indicated a statistically significant difference.

## 3 Results

### 3.1 Baseline Characteristics

A total of 198 patients were included in this study, comprising 151 in the low‐TBS group and 47 in the high‐TBS group. Various baseline characteristics, including age, sex, BMI value, AFP levels, presence of HBV and cirrhosis, portal hypertension, Child–Pugh grade, ablation method, and tumor proximity to large vessels or subcapsular hepatic, were subjected to statistical analysis. Laparoscopic MWA (LMWA) treatment was significantly more prevalent in the high‐TBS group compared to the low‐TBS group (*p* < 0.05). However, there were no statistically significant differences in other indicators between the two groups. Following 1:3 nearest‐neighbor matching analysis, a subset of 44 cases in the high‐TBS group and 95 cases in the low‐TBS group was obtained. This matching process effectively achieved equilibrium in unbalanced covariates between the two groups, with no statistically significant differences observed (*p* > 0.05) (Table 1).

**TABLE 1:** Demographic and clinical characteristics of the enrolled patients before and after 1:3 propensity score matching.

	Before matching			After matching		
Factors	High TBS (*n* = 47)	Low TBS (*n* = 151)	*p*	High TBS (*n* = 44)	Low TBS (*n* = 95)	*p*
Age (years)						
< 60	27	90	0.793	25	54	0.998
≥ 60	20	61		19	41	
Gender						
Male	36	113	0.807	33	71	0.973
Female	11	38		11	24	
BMI						
< 24	25	79	0.917	22	47	0.954
≥ 24	22	72		22	48	
HBV						
Yes	39	132	0.439	36	81	0.605
No	8	19		8	14	
Liver cirrhosis						
Yes	37	118	0.933	34	72	0.848
No	10	33		10	23	
Portal hypertension						
Yes	26	91	0.547	26	55	0.894
No	21	60		18	40	
AFP (ng/ml)						
< 400	37	131	0.180	36	76	0.801
≥ 400	10	20		8	19	
Child–Pugh classification						
A	38	124	0.844	35	78	0.719
B	9	27		9	17	
Ablation method						
PMWA	24	109	**0.007**	24	61	0.277
LMWA	23	42		20	34	
Subcapsular tumor						
Yes	14	34	0.310	13	28	0.993
No	33	117		31	67	
Adjacent to large vessels						
Yes	6	27	0.411	3	15	0.489
No	41	124		39	80	

Abbreviations: AFP: alpha‐fetoprotein; BMI: body mass index; HBV: hepatitis B virus; LMWA: laparoscopic microwave ablation; PMWA: percutaneous microwave ablation; TBS: tumor burden score.

### 3.2 Univariate and Multivariate Survival Analysis

**FIGURE 4:**
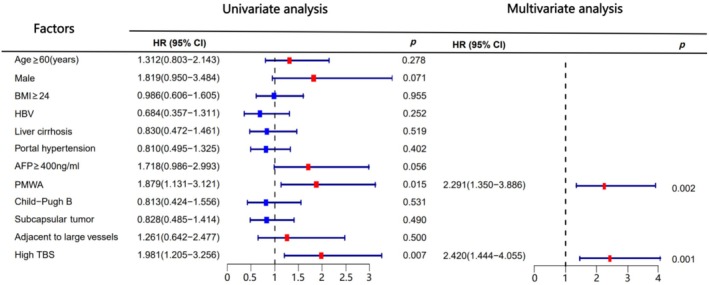
Univariate and multivariate analysis of RFS.

**FIGURE 5:**
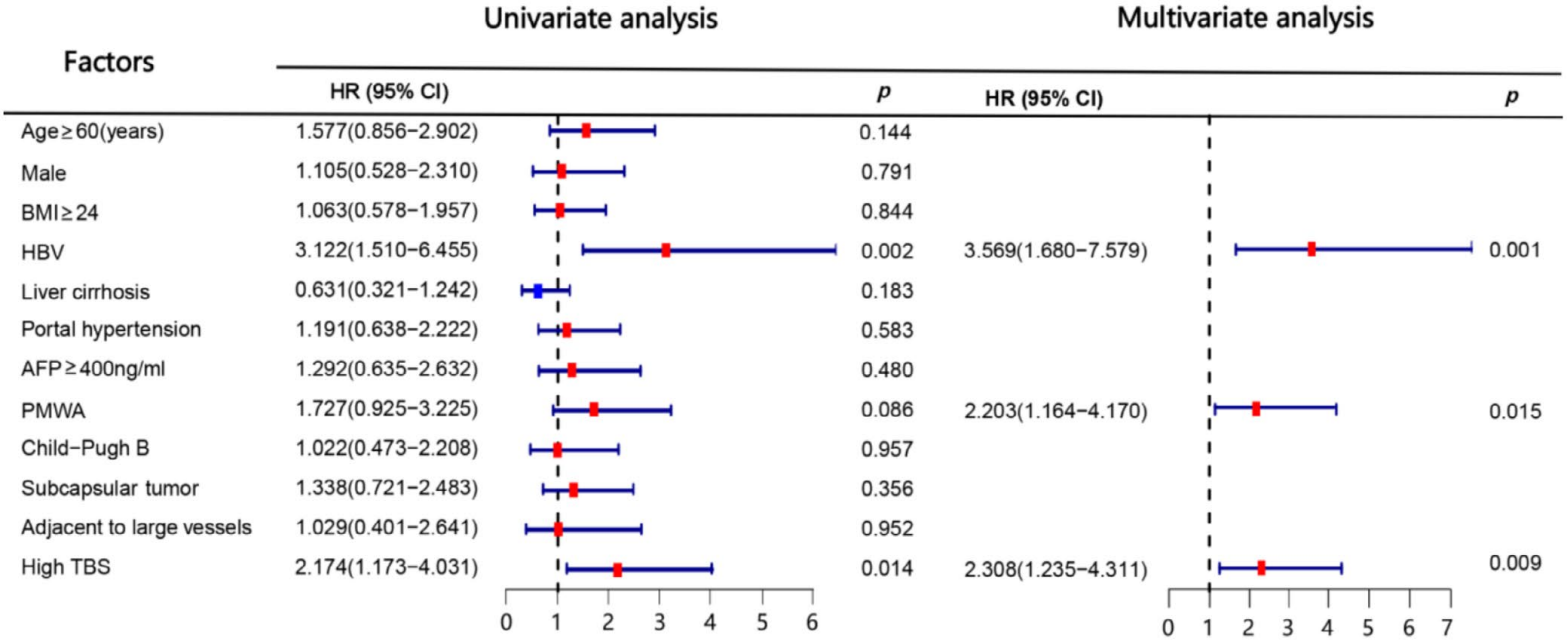
Univariate and multivariate analysis of OS.

The results of the univariate analysis revealed that high TBS and percutaneous MWA (PMWA) were identified as risk factors for recurrence‐free survival (RFS) with statistical significance (*p* < 0.05; Table 2). Additionally, high TBS and the presence of HBV were recognized as risk factors for Overall Survival (OS) (*p* < 0.05; Table 2). In the multivariate analysis, high TBS and PMWA emerged as independent risk factors for RFS (*p* < 0.05; Table 2). Similarly, high TBS, HBV, and PMWA were identified as independent risk factors for OS (*p* < 0.05; Table 2). Notably, variables such as age, sex, BMI, AFP level, cirrhosis, portal hypertension, Child–Pugh grade, and tumor proximity to large vessels and liver capsule did not demonstrate statistically significant correlations with OS and RFS (*p* > 0.05; Table 2). To enhance the visualization effect, we further presented the results of the univariate analysis and multivariate analysis in forest plots in Figures 4 and 5 respectively.

**TABLE 2:** The results of univariate and multivariate analysis using Cox proportional hazards mode.

Factors	Recurrence‐free survival				Overall survival			
	Univariate		Multivariate		Univariate		Multivariate	
	HR (95% CI)	*p*	HR (95% CI)	*p*	HR (95% CI)	*p*	HR (95% CI)	*p*
Age ≥ 60 (years)	1.312 (0.803, 2.143)	0.278			1.577 (0.856, 2.902)	0.144		
Male	1.819 (0.950, 3.484)	0.071			1.105 (0.528, 2.310)	0.791		
BMI ≥ 24	0.986 (0.606, 1.605)	0.955			1.063 (0.578, 1.957)	0.844		
HBV	0.684 (0.357, 1.311)	0.252			3.122 (1.510, 6.455)	0.002	3.569 (1.680, 7.579)	0.001
Liver cirrhosis	0.830 (0.472, 1.461)	0.519			0.631 (0.321, 1.242)	0.183		
Portal hypertension	0.810 (0.495, 1.325)	0.402			1.191 (0.638, 2.222)	0.583		
AFP ≥ 400 ng/mL	1.718 (0.986, 2.993)	0.056			1.292 (0.635, 2.632)	0.480		
PMWA	1.879 (1.131, 3.121)	0.015	2.291 (1.350, 3.886)	0.002	1.727 (0.925, 3.225)	0.086	2.203 (1.164, 4.170)	0.015
Child–Pugh B	0.813 (0.424, 1.556)	0.531			1.022 (0.473, 2.208)	0.957		
Subcapsular tumor	0.828 (0.485, 1.414)	0.490			1.338 (0.721, 2.483)	0.356		
Adjacent to large vessels	1.261 (0.642, 2.477)	0.500			1.029 (0.401, 2.641)	0.952		
High TBS	1.981 (1.205, 3.256)	0.007	2.420 (1.444, 4.055)	0.001	2.174 (1.173, 4.031)	0.014	2.308 (1.235, 4.311)	0.009

Abbreviations: AFP: alpha‐fetoprotein; BMI: body mass index; HBV: hepatitis B virus; PMWA: percutaneous microwave ablation; TBS: tumor burden score.

### 3.3 Survival Analysis

**FIGURE 6:**
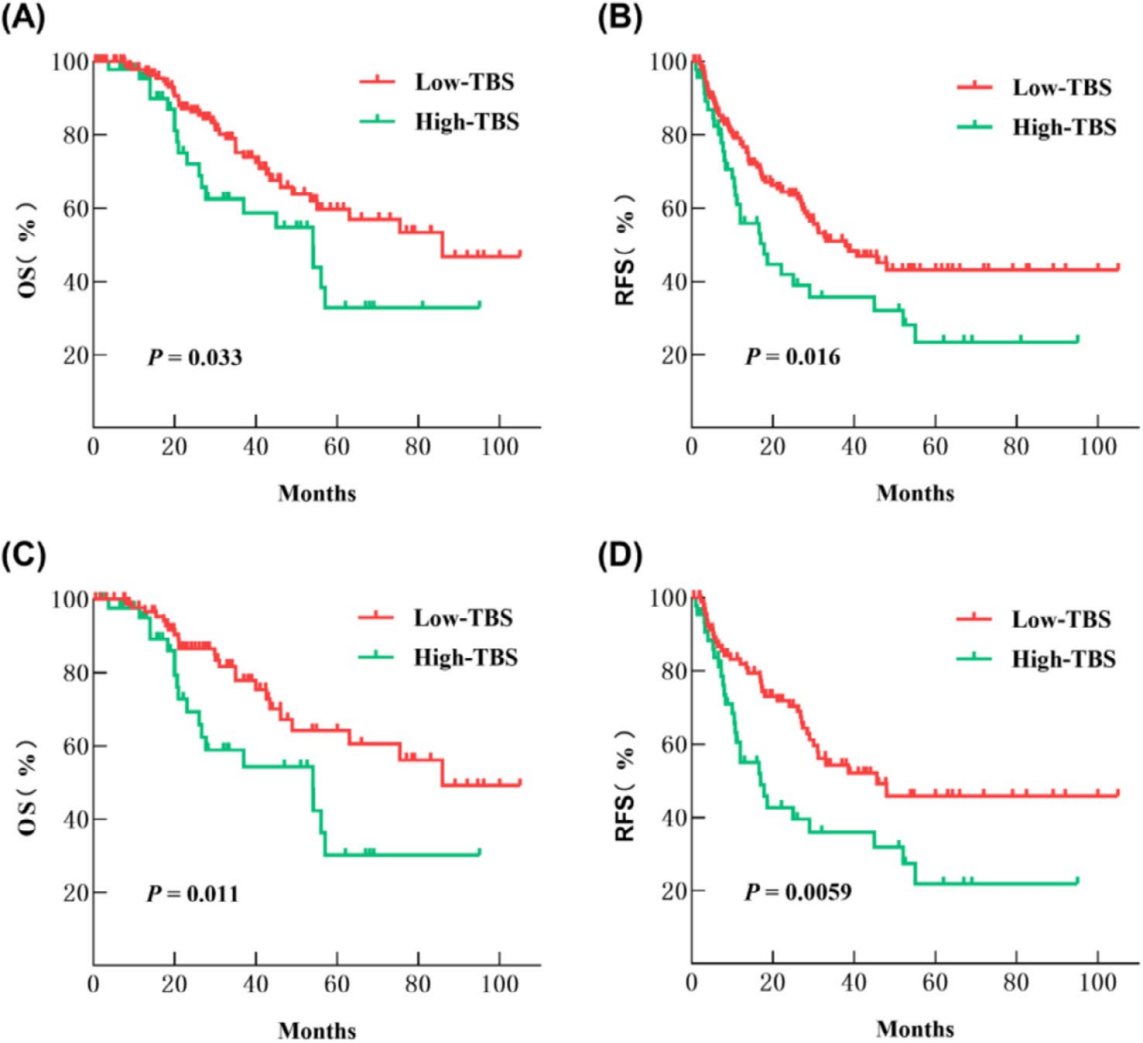
Cumulative OS and RFS of the high‐TBS group and the low‐TBS group before (A and B) and after (C and D) the propensity score matching analysis.

In the pre‐PSM, the median RFS time for the high‐ and low‐TBS groups was 18 and 38 months, respectively. The RFS rates for the two groups at 1 year, 3 years, and 5 years after MWA were 55.8%, 35.7%, and 23.4% for the high‐TBS group, and 77.5%, 50.9%, and 43.1% for the low‐TBS group (*p* = 0.016) (Figure 6B). Similarly, the median OS time for the high‐ and low‐TBS groups was 54 and 86 months, and the OS rates at 1, 3, and 5 years after MWA were 95.3%, 62.6%, and 32.8% for the high‐TBS group, and 97.8%, 75.2%, and 59.7% for the low‐TBS group, respectively (*p* = 0.033) (Figure 6A).

Following PSM, the median RFS time for the high‐ and low‐TBS groups became 17 and 45.6 months, respectively. The RFS rates at 1, 3, and 5 years post‐MWA were 54.9%, 35.9%, and 21.9% for the high‐TBS group, and 81.9%, 54.3%, and 45.9% for the low‐TBS group, respectively (*p* = 0.0059) (Figure 6D). Similarly, the median OS time for the high‐ and low‐TBS groups remained at 54 and 86 months, with OS rates at 1, 3, and 5 years after MWA of 94.9%, 58.9%, and 30.2% for the high‐TBS group, and 97.7%, 77.8%, and 64.1% for the low‐TBS group, respectively (*p* = 0.011) (Figure 6C).

### 3.4 Stratified Analysis of Independent Risk Factors

**FIGURE 7:**
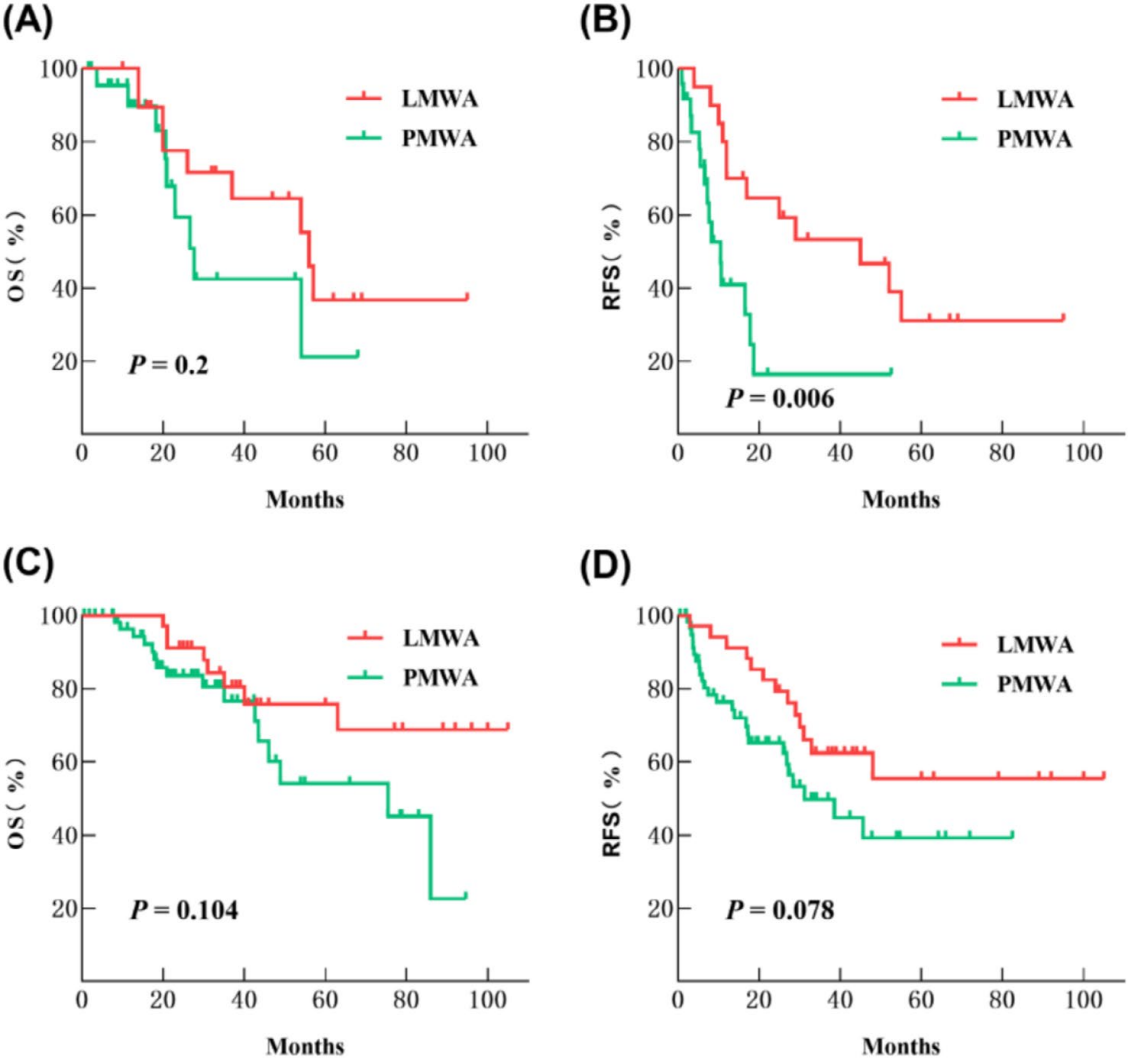
Cumulative OS and RFS of the LMWA and the PMWA in the high‐TBS group (A and B) and the low‐TBS group (C and D) after the propensity score matching analysis.

The stratified analysis conducted on TBS and ablation methods revealed noteworthy insights. Among the 95 patients with low TBS, no statistically significant differences in OS and RFS were found between those treated with LMWA (34 cases) and PMWA (61 cases) (*p* > 0.05) (Figure 7A,B). In the subgroup of 44 patients with high TBS, the median RFS time following LMWA treatment (20 cases) was 45 months, compared to 10.5 months after PMWA treatment (24 cases), demonstrating a statistically significant difference (*p* = 0.006) (Figure 7D). The median OS time was 56 months for LMWA and 27.7 months for PMWA, although no statistically significant difference in OS was observed between the two groups (*p* > 0.05) (Figure 7C).

### 3.5 Complications and Recurrence Rate

The assessment of complications employed the Common Terminology Criteria for Adverse Events (CTCAE) v5.0, as per the National Cancer Institute's guidelines. Among the adverse effects observed, 35.4% (70/198) of patients reported fever, 42.9% (85/198) experienced pain associated with the ablation procedure, and 21.2% (42/198) reported nausea. These effects were predominantly minor (CTCAE Grade 1 or 2) and were effectively alleviated within 2–3 days through the administration of analgesic and antipyretic medications. Only three cases in the high‐TBS group necessitated thoracic puncture drainage (Grade 3), with no other instances of severe complications noted. Postablation enhanced MRI examinations conducted 1 week after the procedure unveiled residual enhancement at the ablation edge in four cases. Of these, three cases belonged to the high‐TBS group and one to the low‐TBS group, all of which achieved complete ablation following remedial ablation procedures. In this study, we categorize the overall recurrence rate to include any form of recurrence, and we specify the local recurrence rate as the recurrence occurring at the site of ablation. At the follow‐up cut‐off point, the overall recurrence rate in the high‐TBS group was 55.3% (26/47), with a local recurrence rate of 27.7% (13/47). In contrast, the low‐TBS group exhibited an overall recurrence rate of 43% (65/151) and a local recurrence rate of 10.6% (16/151). Significantly, a notable difference in the local recurrence rate was observed between the two groups (*p* < 0.05). For patients with recurrence, we promptly implemented treatments such as repeat microwave ablation, TACE (transcatheter arterial chemoembolization), surgical resection, or systemic comprehensive therapy. For patients with intrahepatic metastases, the primary treatments included TACE, microwave ablation, and repeat surgical resection, in conjunction with targeted and immunotherapy. For patients with extrahepatic metastases, systemic comprehensive chemotherapy or radiotherapy was predominantly employed. For those with intrahepatic metastases unsuitable for further resection and ablation, we utilized TACE or radiotherapy, combined with targeted and immunotherapy.

## 4 Discussion

MWA is widely used in the clinical management of early liver cancer, but its association with early postoperative recurrence poses challenges to patient prognosis [4]. Accurate prognostic prediction for liver cancer treated with MWA is vital to identifying high‐risk recurrence patients, optimizing postoperative adjuvant therapy, and minimizing recurrence rates. The conventional dichotomy of BCLC stage and Milan criteria, based on tumor size and number, often constrains the prognostic prediction's statistical power [15, 16]. In response to this challenge, Sasaki proposed a preoperative TBS model designed to convert tumor size and number into a continuous prognostic stratification [8]. This model not only enhances the prediction accuracy of tumor morphological factors but has also demonstrated efficacy in predicting the prognosis of various liver cancer treatments, including surgical resection, liver transplantation, and neoadjuvant chemotherapy for metastatic liver cancer [17, 18]. However, its feasibility and accuracy in evaluating the prognosis of HCC within Milan criteria treated with MWA remain underexplored.

Our study revealed a positive correlation between high TBS and increased local recurrence rates, coupled with a decrease in long‐term survival rates. Ho et al. reported that TBS maintained predictive ability regardless of liver function severity. High‐TBS HCC patients exhibited a 1.4‐ to 2‐fold increased risk of tumor recurrence after ablation and a 1.4–2.1 times elevated risk of reduced OS compared to low‐TBS patients [19]. Another study by Ho et al. demonstrated TBS as a valuable prognostic measure for HCC patients meeting Milanese criteria after radiofrequency ablation (RFA). Moderate and high‐TBS patients had a 37% and 51% increased risk of death, respectively, compared to low‐TBS patients [20]. Administering local chemotherapy before MWA, such as transcatheter arterial chemoembolization (TACE) and hepatic arterial infusion chemotherapy (HAIC), may effectively improve the prognosis of high‐TBS HCC patients [20, 21]. Furthermore, integrating local and systemic therapies, including targeted therapy and immunotherapy, is crucial for high‐TBS and other high‐risk patients to delay recurrence and extend overall survival [22, 23].

In our study, among 44 high‐TBS patients, those treated with LMWA exhibited a significantly prolonged median RFS time compared to PMWA (45 versus 10.5 months, *p* = 0.006). Several factors contributed to this difference: (a) Tumors with higher TBS often have larger diameters and more tumors, increasing the likelihood of microvascular invasion [24]. The integration of intraoperative ultrasound and laparoscopy proved beneficial in not only identifying small lesions that were previously unknown before surgery but also in providing a more flexible grasp of the puncture site and direction. The approach of ablating the adjacent tissues of the tumor from a distance to proximity, progressively moving toward the tumor center, and then executing ablation in a staggered superposition manner demonstrated enhanced efficacy in reducing recurrence compared with PMWA. (b) High‐TBS patients frequently have lesions in specific locations, such as on the surface of the liver, adjacent to the diaphragm, or colon. During percutaneous ablation, subcapsular tumors are susceptible to heat‐induced disintegration and spread. LWMA allowed us to cover the superficial area of the ablation site with wet gauze, reducing the possibility of local diffusion. (c) PWMA, often performed under local anesthesia, may require repeated ablation suspension due to patient discomfort. In contrast, LWMA, performed under general anesthesia, ensures better patient cooperation and a more thorough ablation effect.

This study has several limitations that deserve consideration. First of all, this survey is a retrospective study conducted at a single medical center. Secondly, the sample size is relatively small. After PSM, there are only 44 cases in the high‐TBS group. Thirdly, it must be recognized that all relapsed patients have received additional treatments and medications after MWA, which may affect their survival outcomes. In view of this, future research will focus on conducting multicenter prospective studies. By recruiting more diverse patient groups from different hospitals and regions, the universality and reliability of the research results will be enhanced. Meanwhile, in subsequent studies, the selection criteria for research subjects will be more strictly controlled, and the potential impacts of other treatment measures on survival outcomes will be fully considered and adjusted to evaluate the prognostic value of TBS more accurately.

**FIGURE 8:**
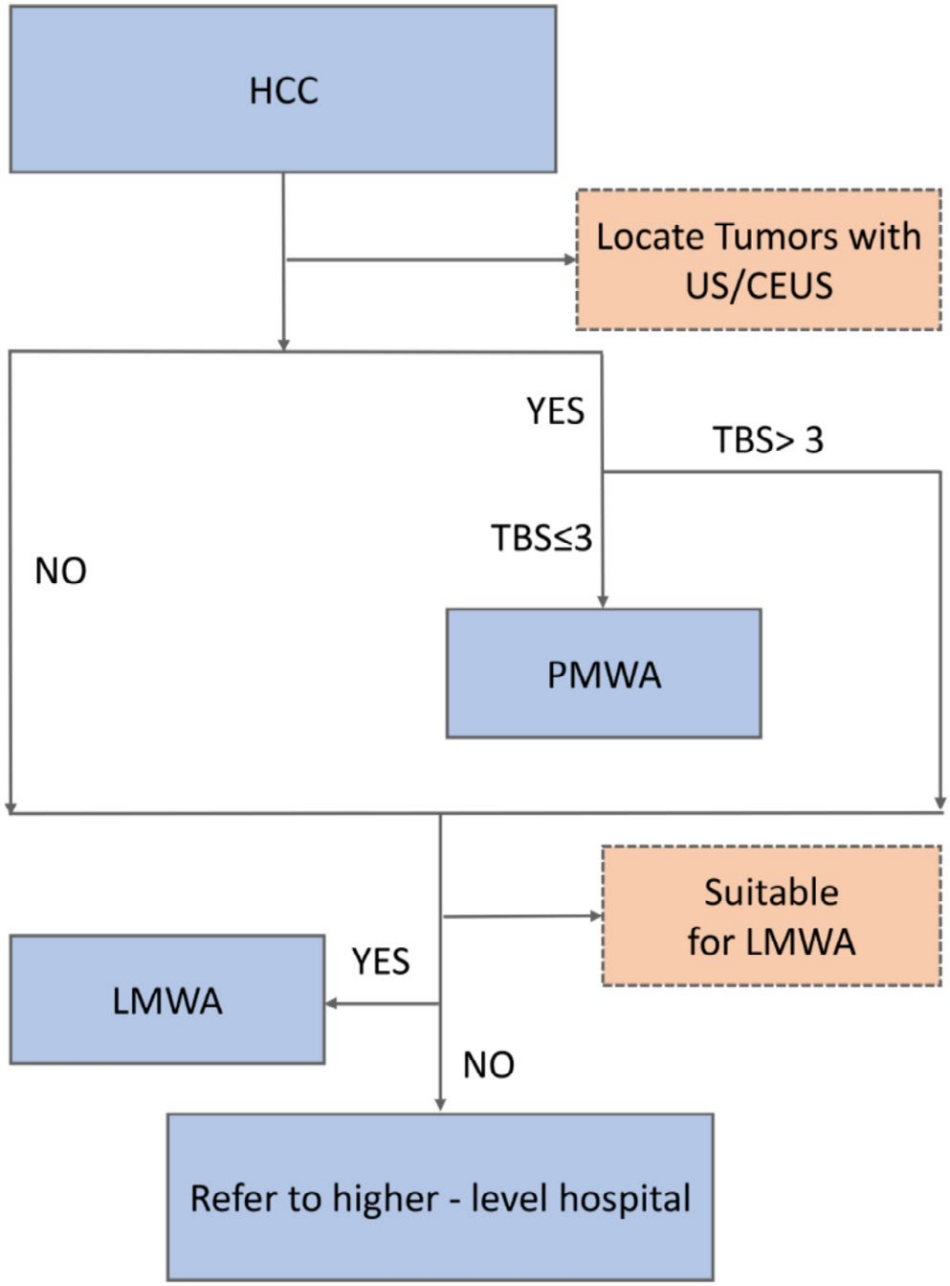
Treatment decision‐making guidance based on TBS.

The advantage of TBS lies in its simple calculation. It can be obtained through ultrasound even in primary hospitals with limited resources. Thus, we've carefully devised relevant diagnostic and treatment protocols, shown in Figure 8 for primary—level medical institutions' reference. When ultrasound can precisely locate the tumor and TBS ≤ 3, primary hospitals are advised to use the PMWA treatment. If the tumor can't be located by ultrasound or TBS > 3, hospitals should decide on the LMWA treatment plan based on their laparoscopic skills and the patient's condition. Otherwise, the patient must be promptly referred to a higher –level hospital. The results of this study have a certain supplementary effect on the existing guidelines based on the Milan criteria. The inclusion of TBS can refine the risk stratification. For patients who meet the Milan criteria but have a TBS > 3, their recurrence risk and poor prognosis increase. It is recommended to shorten the follow –up interval for such patients. In terms of treatment selection, as this study found that LMWA has an advantage in recurrence –free survival among patients with a TBS > 3 within the Milan criteria, the guidelines can prioritize the recommendation of LMWA after considering the individual situation of the patient.

In addition, in this study, the selection of the specific TBS cut‐off value (TBS > 3) is based on our data analysis, aiming to maximize the discriminatory ability between groups. Although this cut‐off value is statistically significant in our study population, differences in patient characteristics and imaging techniques among different centers may exist, which may affect the optimal cut‐off value. Future multicenter studies with larger sample sizes are needed to further validate and refine the TBS cut‐off value. Therefore, we recommend that clinicians interpret TBS results in combination with other clinical factors and consider it as part of a comprehensive assessment rather than relying solely on this cut‐off value.

## 5 Conclusion

In summary, TBS proves to be a valuable predictor for the long‐term outcomes of HCC patients meeting Milan criteria after MWA. A higher TBS is associated with a lower long‐term survival rate. Notably, among HCC patients meeting Milan criteria, those with TBS > 3 exhibited an extended median RFS time after LMWA treatment.

## Author Contributions

**Xiaolin Liu:** funding acquisition (lead), writing – original draft (lead), writing – review and editing (lead). **Jing Wang:** project administration (equal), resources (equal). **Feng Xu:** data curation (lead), investigation (lead), methodology (lead). **Jing Chen:** data curation (equal), formal analysis (lead), investigation (equal). **Mingyuan Zhu:** project administration (equal), resources (equal). **Xiaoguang Wang:** project administration (lead), resources (lead).

## Ethics Statement

The studies involving human participants were reviewed and approved by the Medical Ethics Committee of The Second Affiliated Hospital of Jiaxing University (2023SW001‐01).

## Consent

Written informed consent was obtained from the individuals for the publication of any potentially identifiable images or data included in this article.

## Conflicts of Interest

The authors declare no conflicts of interest.

## Clinical Trial Registration

The authors have nothing to report.

## Permission to Reproduce Material From Other Sources

The authors have nothing to report.

## Data Availability

Data is available on request from the authors.
